# A *TUBB4A* Met363Thr variant in pediatric hypomyelination without atrophy of the basal ganglia

**DOI:** 10.1038/s41439-022-00198-6

**Published:** 2022-06-03

**Authors:** Marina Hashiguchi, Yukifumi Monden, Yasuyuki Nozaki, Kazuki Watanabe, Mitsuko Nakashima, Hirotomo Saitsu, Takanori Yamagata, Hitoshi Osaka

**Affiliations:** 1 Department of Pediatrics, Jichi Medical University, Tochigi, Japan; 2 Department of Pediatrics, Shin-Oyama City Hospital, Tochigi, Japan; 3 Department of Biochemistry, Hamamatsu University School of Medicine, Shizuoka, Japan

**Keywords:** Neurodegeneration, Neurodegeneration

## Abstract

*TUBB4A* gene variants cause dystonia type 4 and hypomyelination with atrophy of the basal ganglia and cerebellum. We report the case of a child with delayed motor development, intellectual disability, and dystonia. Magnetic resonance imaging revealed hypomyelination and progressive cerebellar atrophy without atrophy of the basal ganglia. Whole-exome sequencing revealed a de novo heterozygous variant, c.1088T > C, p.(Met363Thr), in *TUBB4A*. The present case further supports the vulnerability of the cerebellum in patients with *TUBB4A* pathogenic variants.

Hypomyelination with atrophy of the basal ganglia and cerebellum (H-ABC) is caused by heterozygous variants in *TUBB4A*^1,2^. Variants in *TUBB4A* are known to cause two different clinical conditions: dystonia type 4 (DYT4) and H-ABC^1,3^. The DYT4 phenotype is characterized by whispering dysphonia, generalized dystonia, and gait ataxia. Magnetic resonance imaging (MRI) typically reveals a normal brain findings^3^. Patients with H-ABC exhibit clinical onset in early infancy, with developmental delay, extrapyramidal symptoms, progressive spastic tetraplegia, ataxia, dysarthria, cognitive and sensory deficits, and seizures^1^. Characteristic MRI findings include white matter hypomyelination, the absence or disappearance of the putamen, and cerebellar atrophy^1^.

There is great diversity in the age of onset, clinical course, and brain MRI findings associated with *TUBB4A* variants^4,5^. In particular, there are a series of *TUBB4A*-associated phenotypes with isolated hypomyelination that do not fit either DYT or H-ABC patterns^4–18^.

We describe the case of a patient with *TUBB4A*-related hypomyelination caused by a c.1088T > C, p.(Met363Thr) variant. This variant has been reported once previously, but no details of the course of the disorder were available^19^. The following report presents the case of this patient with *TUBB4A*-related hypomyelination and cerebellar atrophy without atrophy of the basal ganglia.

The patient was an 11-year-old boy who presented with a neurodevelopmental disorder. His family and perinatal histories were unremarkable. He first spoke at 12 months of age and began walking independently at 18 months of age. From 3 years of age, he was noted to be clumsy and unsteady and exhibited difficulties with comprehension. He attended both special and regular education classes in elementary school. At the age of 10 years, he was diagnosed with mild intellectual disability (IQ = 52). Physical examination revealed no abnormalities. However, the neurological examination found brisk deep tendon reflexes, a bilateral Babinski sign, ankle clonus, upper limb dystonia, and abnormal tandem gait. MRI at the age of 8 years revealed cerebral white matter hypomyelination but no atrophic findings in the basal ganglia or cerebellum (Fig. 1A). MRI at the age of 11 years revealed hypomyelination and atrophy of the cerebellum, but the basal ganglia size was normal (Fig. 1B, C). Whole-exome sequencing was performed, and the NM_006087.4: c.1088T > C, p.(Met363Thr) variant was detected. Since neither parent had this variant, it was considered a de novo variant. This variant was absent in gnomAD v3.1.2 (accessed January 2022), the ToMMo 14KJPN Allele Frequency Panel (v v20211208) (https://jmorp.megabank.tohoku.ac.jp/202112/), and 218 in-house Japanese exome control datasets. In silico evaluation tools predicted this variant to be deleterious (PROVEAN −3.42, CADD v1.6 phred 23.8, M-CAP 0.869). Based on the American College of Medical Genetics and Genomics standards and guidelines, the c.1088T > C variant was classified as likely pathogenic (PS2, PM2, PP3).

**Fig. 1:**
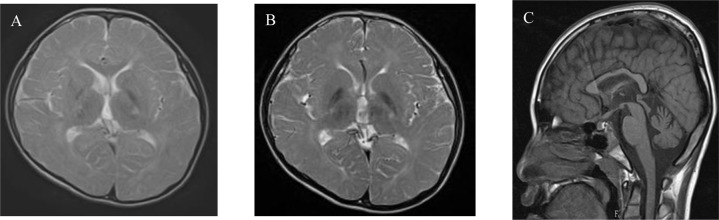
Brain MRI images. **A** Axial T2 image at the age of 8 years shows diffuse hypomyelination without atrophy of the basal ganglia. **B** Axial T2 image at the age of 11 years shows persistent hypomyelination without atrophy of the basal ganglia. **C** Sagittal T1 image at the age of 11 years shows atrophy of the cerebellum.

*TUBB4A* variants causing H-ABC were first reported in 2013, with an onset age between 2 months and 4.5 years (median age: 6 months)^8^. The frequency of atrophy of the basal ganglia in H-ABC has been reported to be 70% in the capsule and 30% in the caudate nucleus within 2 years of onset but progresses to 97% and 53% (2–12 years after onset) and then 100% and 90%, respectively, (>12 years after onset)^20^. However, some patients with hypomyelination but without atrophy of the basal ganglia have been reported to have isolated hypomyelination^4,18^. We summarize the variants of *TUBB4A*-related hypomyelination without atrophy of the basal ganglia (H-without AB) in Table 1. The age at onset of clinical symptoms was between 1 month and 33 years (median age: 15 months), which is later than that for H-ABC. Including the present case, cerebellar atrophy was reported with 12 of the 17 variants (70%) (Table 1).

**Table 1:** Summary of previously reported *TUBB4A* variants of H-without AB.

	Nucleotide change	Protein change	MRI			Age at onset	Reference
			Hypomyelination	Cerebellar atrophy	basal ganglia atrophy		
H-ABC			+	+	+		
DYT-4			−	−	−		
	c.286G > A	p.G96R	+	−	−	17 Y	Lu^8^
	c.467G > T	p.R156L	+	+	−	2 m	Purnell^5^
	c.533C > T	p.T178M	+	+	−	20 m	Tonduti^10^
	c.535G > C	p.V179L	+	−	−	2 m	Isakov^13^
	c.539T > G	p.V180G	+	−	−	1 Y	Vanderver^11^
	c.544C > A	p.P182T	+	+	−	6 m	Tonduti^10^
	c.568C > T	p.H190Y	+	+	−	1−18 m	Kancheva^14^
	c.731G > A	p.G244D	+	±	−	6−30 m	Tonduti^10^
H-without AB	c.763G > A	p.V255I	+	+	−	2 Y	Curiel^4^
	c.785 G > A	p.R262H	+	+	−	0 Y	Ferrira^16^
	c.845G > C	p.R282P	+	+	−	2 Y, 5 Y	Curiel^4^
	c.874C > A	p.Q292K	+	−	−	3 Y	Pizzino^18^
	c.900G > T	p.M300I	+	+	−	1−30 Y	Pyle^15^, Erro^12^
	c.1064A > T	p.D355V	+	+	−	Childhood, 33 Y	Sagnelli^9^, Bella^6^
	c.1088T > C	p.M363T	+	+	−	1 Y	The present case
	c.1172G > A	p.R391H	+	−	−	1 Y, childhood	Pizzino^18^, Vanderver^11^
	c.1242C > G	p.N414K	+	+	−	−	Duncan^7^
						0 Y−33 Y (median: 15 m)	

Several correlations between genotype and phenotypic severity in H-ABC have been suggested. Patients with the common c.745G > A variant have a more benign phenotype than patients with other variants^20^. Lu et al.^8^ reported that variants located on the outside of the αβ-tubulin heterodimer, distant from the guanosine triphosphate domain, are likely to result in milder phenotypes without atrophy of the basal ganglia or cerebellum. For the c.900G > T, c.1064A > T, and c.1172G > A variants, there have been two reports of phenotypes characterized by hypomyelination without atrophy of the basal ganglia (Table 1). However, Tonduti et al.^10^ reported that patients with the same variant showed different disease courses.

To our knowledge, there has been no report on *TUBB4A*-related hypomyelination with only atrophy of the basal ganglia. Therefore, we speculate that the cerebellum is more vulnerable than the basal ganglia. The highest expression of *TUBB4A* was in the cerebellum, followed by the putamen and white matter^3^. There was a twofold difference between the cerebellum and thalamus, which had the lowest expression^3^. The different *TUBB4A* expression levels in different brain regions may explain this distinct vulnerability.

## HGV Database

The relevant data from this Data Report are hosted at the Human Genome Variation Database at 10.6084/m9.figshare.hgv.3199.
